# FracAtlas: A Dataset for Fracture Classification, Localization and Segmentation of Musculoskeletal Radiographs

**DOI:** 10.1038/s41597-023-02432-4

**Published:** 2023-08-05

**Authors:** Iftekharul Abedeen, Md. Ashiqur Rahman, Fatema Zohra Prottyasha, Tasnim Ahmed, Tareque Mohmud Chowdhury, Swakkhar Shatabda

**Affiliations:** 1 Islamic University of Technology, Gazipur, 1704 Bangladesh; 2 United International University, Dhaka, 1212 Bangladesh

**Keywords:** Medical research, Diagnosis

## Abstract

Digital radiography is one of the most common and cost-effective standards for the diagnosis of bone fractures. For such diagnoses expert intervention is required which is time-consuming and demands rigorous training. With the recent growth of computer vision algorithms, there is a surge of interest in computer-aided diagnosis. The development of algorithms demands large datasets with proper annotations. Existing X-Ray datasets are either small or lack proper annotation, which hinders the development of machine-learning algorithms and evaluation of the relative performance of algorithms for classification, localization, and segmentation. We present FracAtlas, a new dataset of X-Ray scans curated from the images collected from 3 major hospitals in Bangladesh. Our dataset includes 4,083 images that have been manually annotated for bone fracture classification, localization, and segmentation with the help of 2 expert radiologists and an orthopedist using the open-source labeling platform, makesense.ai. There are 717 images with 922 instances of fractures. Each of the fracture instances has its own mask and bounding box, whereas the scans also have global labels for classification tasks. We believe the dataset will be a valuable resource for researchers interested in developing and evaluating machine learning algorithms for bone fracture diagnosis.

## Background & Summary

There has been a surge in demand for computer-aided diagnosis (CAD) systems in recent decades. Moreover, recently different fields of medical science have seen rapid development of automation processes in diagnosis leveraging large datasets and advanced machine learning algorithms^1,2^. Models like convolutional neural networks (CNN)^3^, You only look once (YOLO)^4^ and U-NET^5^ can achieve expert-like performance in detecting anomalies from X-Ray scans. Training such models requires large and well-annotated datasets^6–8^. It is difficult to collect such data from hospitals and diagnostic centers. The annotation process can be very costly as it requires the involvement of multiple physicians and radiologists for consensus to remove bias and human errors. Due to the sensitive nature of medical data, it is also very hard to make the acquired data available for public use. To sum it up the creation of such datasets is costly and time-consuming^2,9^.

Some of the well-known X-Ray datasets that are publicly available and that focus on anomalies include MURA^10^, MedPix^11^, GRAZPEDWRI-DX^12^, IIEST^1^, MOST^13^, VinDr-CXR^2^, VinDr-SpineXR^14^ and ChestX-ray14^15^. Among these datasets, MURA is a collection of 2D muscular skeletal radiographs with 40,561 images from different regions such as the elbow, finger, forearm, hand, humerus, shoulder, and wrist^10^. Each image is labeled as ‘Normal’ or ‘Abnormal’ which makes it suitable for classification tasks, however, it lacks proper annotation for localization and segmentation. MedPix is an online database of 2D and 3D medical scans of various diseases that can be filtered by the keyword ‘fracture’ resulting in 954 images. These images include X-rays, real images, Magnetic resonance imaging (MRI), Computed tomography (CT) scans, and ultrasound imaging. However, the dataset has issues such as unorganized annotation and falsely labeled images, as well as some spam images. GRAZPEDWRI-DX is a recently released dataset, containing 20,327 scans with annotation for localization collected from 6,091 patients. Though this is a suitably large dataset, it covers only wrist fractures, omitting the rest of the parts of the human body. IIEST is a small dataset of 2D X-rays containing 217 images, of which 49 are healthy, 99 are fractured and 69 are cancerous bone scans. This dataset is very small and inadequate for machine-learning activities. MOST is a dataset that contains 4,446 X-ray and MRI scans labeled by the Kellgren–Lawrence (KL) grading system^16^ having five classes from grade-0 to grade-4 with increasing severity from one to the next. This dataset is no longer available in the public domain due to lacking of funding and closeout. It also covers only knee joint fractures. VinDr-CXR is also a recently published dataset, which contains 18,000 images of chest X-rays (CXR) with manual annotation for localization. This dataset contains samples for 28 different types of chest diseases and abnormalities. Though this is a good dataset for identifying chest diseases, it’s not suitable for bone fracture identification. Likewise, the VinDr group has other datasets VinDr-Mammo^17^, VinDr-SpineXR^14^ and PediCXR^18^ which are not suitable for fracture study for similar reasons. ChestX-ray14 is a dataset for radio graphs containing 112,000 CXR scans. This dataset is also not suitable for bone fracture study as it only contains samples of chest diseases. Some prominent datasets of human body radiographs are compared with the FracAtlas dataset^19^ in Table 1.

**Table 1:** An overview of existing X-ray datasets.

Dataset	Release year	samples	Global labels	Local labels	Local mask	Multi-locale
MURA^10^	2017	40,561	Available	N/A*	N/A	Yes
MedPix^11^^††^	2016	1,954	Available	N/A	N/A	Yes
GRAZPEDWRI-DX^12^^†^	2022	20,327	Available	Available	N/A	No
IIEST^1^ ^(Δ)^	2020	217	Available	N/A	N/A	Yes
MOST^13^	2020	4,446	Available	N/A	N/A	Yes
VinDr-CXR^2^ (•)	2022	18,000	Available	Available	N/A	No
PediCXR^17^ (•)	2023	9,125	Available	Available	N/A	No
ChestX-ray14^15^ (•)	2017	112,120	Available	N/A	N/A	No
RSNA Pediatric^26^ (•)	2017	14,236	Available	N/A	N/A	No
FracAtlas	2023	4,083	Available	Available	Available	Yes

*Can be generated or interpreted from the given data. ^(†)^Used a mixture of automated NLP tools and Manual labeling. ^(††)^Annotation is fully automated without any manual validation. ^(Δ)^Too small to be applicable for training deep learning models. ^(•)^Not suitable for fracture study.

The shortcomings of the existing datasets are that most of them can only be used for classification tasks or they lack proper annotation. Some are also mislabeled and hence not suitable for machine learning tasks as they are not well maintained or the quality of annotation is not up to the mark. The available high-quality X-ray datasets are not intended for bone fracture study. With the recent advancements in CAD systems, datasets for only classification tasks are not enough as most are moving toward developing localization and segmentation models^20^. For such tasks, it is very important to have well-maintained and documented datasets with proper manual annotation. Due to the sensitive nature of the medical domain, it is very important for the models to perform at a high level. And to accomplish that, a large dataset with high-quality annotation is very important^7^.

Most of the prominent works on bone fracture classification, localization and segmentation have used private datasets^21–23^. Due to the unavailability of publicly accessible datasets currently, it is not feasible to conduct a comparative analysis of state-of-the-art (SOTA) methods. To solve this problem we introduce FracAtlas dataset^19^ which has been created by collecting 14,068 x-ray scans from three prominent hospitals in Bangladesh. From these 14,068 scans, 4,083 images have been isolated from regions like hand, shoulder, leg and hip. The rest of the scans were discarded as they were from the chest or skull region. Due to security and privacy concerns, we have anonymized all the patient-related structured data such as name, age, gender, time of diagnosis, etc. from each of the scans. The collected DICOM images have been converted to JPG format. The dataset can be accessed at figshare (10.6084/m9.figshare.22363012).

## Methods

**Fig. 1:**
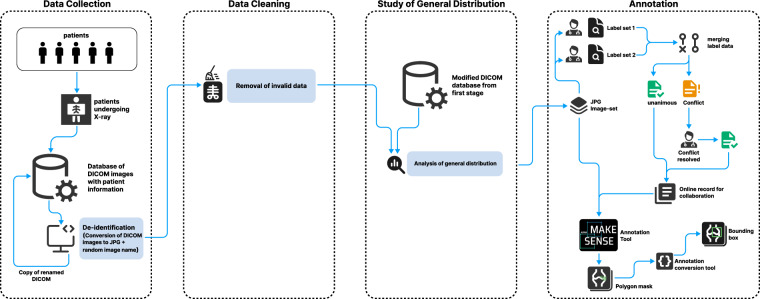
The workflow for creating the FracAtlas dataset: (1) general purpose X-ray images were collected in DICOM format and for de-identification, the images were converted to JPG and were given arbitrary names. (2) The resultant JPG image set from Stage 1 was filtered out from other body parts. (3) The resulting image set from stage 2 was taken back to the respective hospitals to find out the general distribution. (4) the resulting image set from stage 2 was annotated by 2 expert radiologists and later verified and merged by an expert orthopedic doctor. Masks were developed manually with open-source software based on the generated labels by the doctor in COCO JSON format. The resulting masks were then converted to other annotation formats for use in different machine-learning purposes.

We have created the FracAtlas dataset^19^ in four main steps (1) Data Collection (2) data cleaning (3) finding the general distribution of cleaned data (4) annotation of the dataset. Throughout the years 2021 and 2022, approximately 14,068 X-ray scans were collected from 3 hospitals and diagnostic centers. Most of the scans were collected from Lab-Aid Medical Center, Brahmanbaria, along with Anupam General Hospital and Diagnostic Center, Bogra and Prime Diagnostic Center, Barishal. The acquired DICOM images were generated by Fujifilm and Philips devices. The complete process is illustrated in Fig. 1. The ethical clearance of this study was approved by Institutional Research Ethics Board (IREB) according to the Bangladesh Medical Research Council (BMRC). The IREB approved the open publication of the data based on the facts that there are adequate provisions to maintain the confidentiality of the individuals through proper filtration of personally identifiable information. Furthermore, the permission of publishing the data to the public domain was also taken at the source. Consent for data collection for all subjects (adults and parents in the case of minors) was taken as part of the initiation of the diagnosis at the medical facilities. Also, the data collection process had no effect on the clinical treatment or processes of diagnosis of the three hospitals involved and all personally identifiable information in the gathered data has been removed. The whole process was administered according to the Institutional Research Ethics Board of United International University.

### Data collection and cleanup

**Fig. 2:**
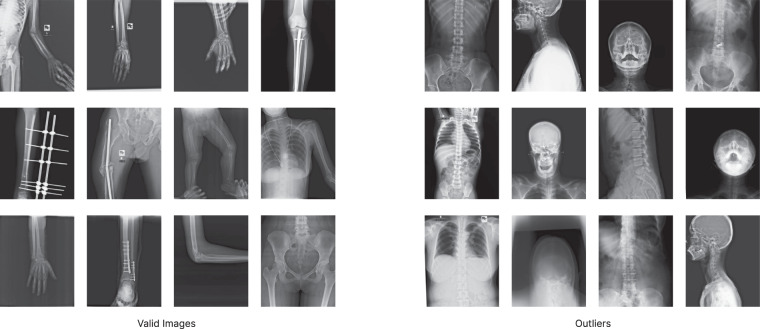
Example of valid (Left) vs outlier (Right) X-ray images. All the scans were manually filtered based on the parts of the body present in the scan, clarity of the scans and resolution. The scans containing only arm, shoulder, leg and hip regions were accepted.

In the initial phase, a total of 14,068 X-Rays were collected. As the hospitals and diagnostic centers could not share patient information due to privacy concerns, all the DICOM images were given an arbitrary image name and converted to JPG image format. This automatically got rid of all the sensitive information that was present in the metadata of DICOM images. These conversions were done using the proprietary software of the corresponding X-ray machines. The renaming process was automated using a Python script. The renamed DICOM images were stored in the hospital database separately for later study of general distribution. All the X-ray scans that have been collected are for general-purpose diagnosis. This means along with bone fracture scans there are also samples for chest diseases and abnormalities in the skull and spine region. In the collected data the number of bone fracture samples in the chest, skull and spine region was sparse. As a result, scans for the said parts were removed with the supervision of a medical officer. This left us with 4,083 scans from the hand, leg, hip and shoulder regions. Figure 2 shows some valid vs outlier images for the dataset. Some of the images in our dataset contain logos and texts which have not been removed.

### Distribution analysis

**Fig. 3:**
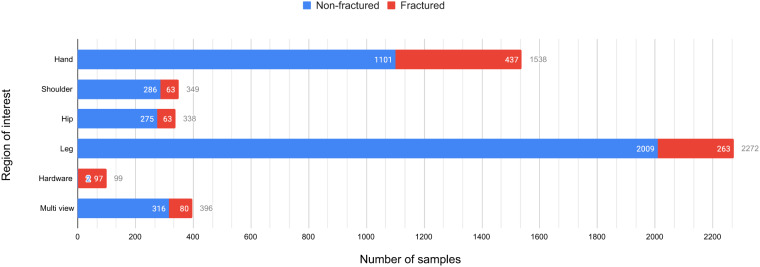
The distribution of different locales along with other properties present in the images of the FracAtlas dataset. The locales include the hand, shoulder, hip and leg region. The distributions also show the number of Orthopedic Fixation Devices (Hardware) and images with a split view (Multi-view) of the same organs from different planes. The blue chart shows the distribution of the said attributes for the healthy scans of the dataset where the red bars show the same for fractured scans and they collectively show the overall distribution for the whole dataset. The numbers right of each bar with the corresponding color represent the value, whereas the gray numbers represent the collective values.

**Fig. 4:**
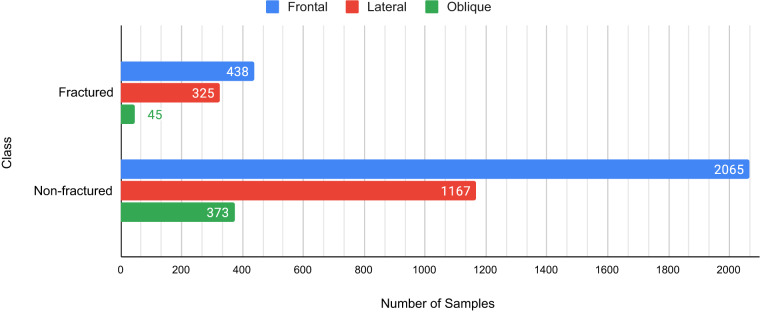
The number of samples present for each of the frontal, lateral and oblique views present in the FracAtlas dataset for individual classes.

After the cleanup process, the demographic analysis was done on the 4,083 images. In our study, we have observed that the age of the patients has a major impact in terms of fracture analysis. For younger subjects (0–7 years old) the ends of bones near the joints can look like separate disjoint disc pads due to lack of bone density. A model trained on patients above this said range can misjudge those bone structures as fractures. On the contrary, for older patients (above 50 years old) the surface of bones can look rough^24^. This can also lead a model to misjudge those bones as fractured ones. So, it is crucial that a dataset intended for fracture study contains a diverse range of patients’ ages. As all metadata of the X-Ray images were discarded at the time of collection. After dataset cleanup, the remaining images were taken back to the corresponding hospitals to find out the distribution of age and gender on the entire dataset. The age of subjects in our dataset ranges from 8 months to 78 years old. Also, the gender distribution for abnormal studies is 85.4% and 14.6% between males and females respectively. The gender ratio for the whole dataset (normal + abnormal cases) is 62% male and 38% female approximately. There are 717 abnormal scans in our dataset which contain a total of 922 instances of fractures. The abnormal studies contain at least 1 and at most 5 fracture instances in them. Some of the scans have multiple views and locales in them. The whole dataset contains 4,083 images and 4,497 locales. There are 396 images with different views of the same organ in the same image. There are 99 images with Orthopedic Fixation Devices (hardware) in them. The FracAtlas dataset^19^ has a total of 1,538 scans of the hand and among them, 437 are fractured. There is a total of 2,272 leg scans, 338 hip scans and 349 shoulder scans. Among these, the number of scans belonging fractured class is 263, 63 and 63 for the leg, hip and shoulder regions respectively. Figure 3 illustrates the distribution of different properties present in the dataset. The FracAtlas dataset comprises a total of 2,503 frontal, 1,492 lateral, and 418 oblique view images, each pertaining to different organs. Whereas the ‘Fractured’ class includes 438 frontal, 325 lateral, and 45 oblique view images. Conversely, the ‘Non-fractured’ class encompasses a total of 2,065 frontal, 1,167 lateral, and 373 oblique views. The relative distribution is illustrated in Fig. 4.

### Data labeling

**Fig. 5:**
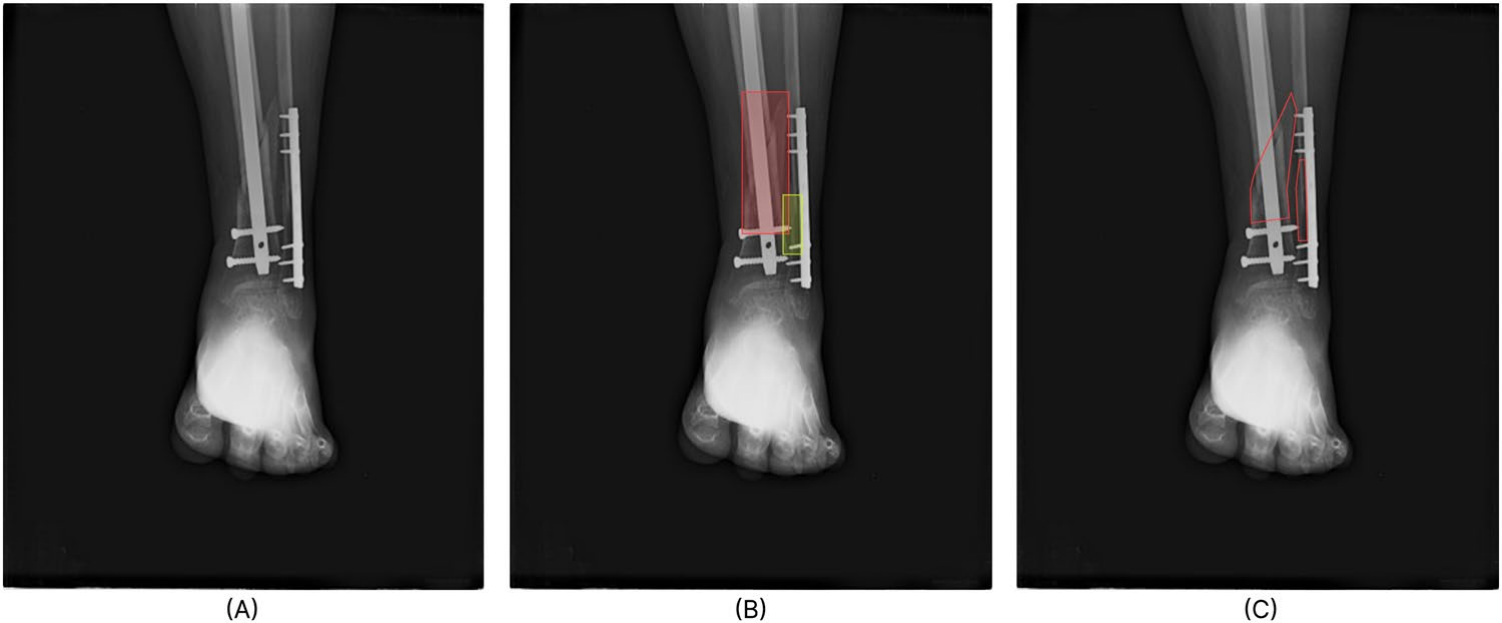
Fully tagged and labeled sample image. (**A**) shows the original scan with global tags leg, hardware, fractured set to 1 (true) and fracture count set to 2. The remaining tags (hand, hip, shoulder, mixed, multiscan) are set to 0 (false) (**B**) The boxes mark the local region of the fracture instance for localization tasks. (**C**) The red borders mask the fracture regions for segmentation tasks.

The distribution analysis of the data was followed by a review process by two expert radiologists, each with years of experience in the field. The radiologists went through all 4,083 images and labeled each image by identifying the presence and number of fractures, along with the location name of the fractures. After full observation, the fracture list generated by each radiologist was cross-checked with one another. The images that had unanimous labels provided by the radiologists were taken as fractured scans. In case of any disparities in the location of fractures or the count of fracture locales, the images were referred to an expert Orthopedic surgeon for further review and validation. After labeling those listed images independently, the images were again cross-checked with his own findings to the ones generated by the radiologists. And after comparing all 3 samples the final labels were agreed upon. After resolving all conflicts, the images were manually annotated using makesense.ai https://github.com/SkalskiP/make-sense. The primary type of annotation generated for bone fracture was in Common Objects in Context (COCO) format^25^. This format allows for the creation of polygon masks of the fracture regions. Each image can have multiple locales marked by separate masks and different masks are also allowed to overlap. The COCO JavaScript Object Notation (JSON) format was chosen to be worked on manually because it contains the most amount of information and allows conversions that are lossy to other annotation formats like YOLO annotation and Pascal VOC and also lossless ones like Visual Geometry Group (VGG) format. The COCO JSON format is used for segmentation tasks whereas YOLO and Pascal Visual Object Classes (VOC) are used for localization. The original record maintained for the primary labeling process is also provided for classification tasks in Comma Separated Values (CSV) format. Figure 5 shows different annotation types provided with the dataset.

## Data Records

The FracAtlas dataset^19^ has been made available for public download through Figshare 10.6084/m9.figshare.22363012). The data can be downloaded without any need for registration. The total size of our dataset is 323 MB. The folder structure containing the dataset with all relevant files is described below.

### Folder structure

**Fig. 6:**
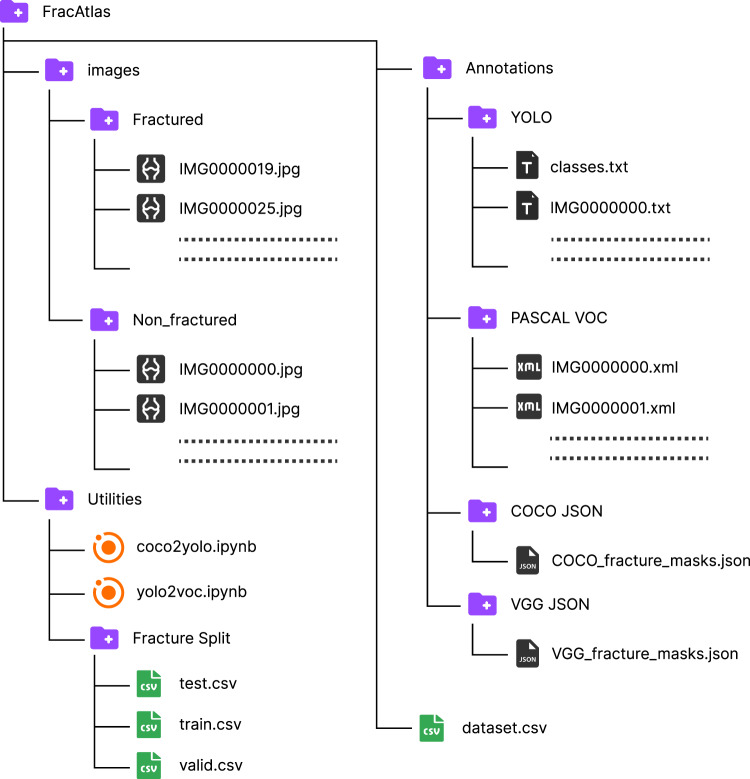
The folder structure of the FracAtlas dataset.

The root folder for the dataset is named “FracAtlas”. It contains subfolders “images”, “Annotations”, “utilities” and a “dataset.csv” file. The CSV file has been generated during the data labeling process discussed in the methods section. Figure 6 gives an overview of the folder structure. The CSV contains columns representing whether a scan has “hand”, “leg”, “hip” or “shoulder” region present in it along with the information if the scan contains multiple regions in the scan. It also has a “hardware” column corresponding to the availability of Orthopedic Fixation Devices in the scan. Some X-ray scans have multiple views of the same organ projected from the frontal (Coronal) plane and Sagittal plane. Those images can be identified using the “multiscan” column in the CSV. The “fractured” column represents if a scan has fractures in it. All the column mentioned so far has binary value containing ‘0’ and ‘1’. The ‘0’ and ‘1’ represents a specific attribute being absent or present in that particular image respectively. The only exception to this is the “fracture_count” column which has numerical values from 0 and 5 representing the number of fracture instances present in that image. The “frontal”, “lateral” and “oblique” columns represent the perspectives present in a scan with values set to ‘1’ and ‘0’ otherwise.

The “images” folder has two subfolders named “Fractured” and “Non_fractured”. The “Fractured” Folder contains all the images containing fractures in them. Whereas the Folder “Non_fractured” contains all the healthy bone radiographs. All the scan names start with “IMG” followed by zero padded seven-digit values which are unique to each image ending with “.jpg” signifying the datatype. The folder named “Annotation” comprises various annotation files for segmentation and localization purposes. For segmentation, there are two subfolders named “COCO JSON” and “VGG JSON”, which contain corresponding annotation types in the “.json” format. These files contain annotations only for images that have fractures. For localization, there are two subfolders named “YOLO” and “PASCAL VOC” (PASCAL Visual Object Classes) containing “.txt” and “.xml” files, respectively, named after the corresponding image files. Additionally, the “YOLO” folder has a “classes.txt” file that lists the available classes for localization, and in this case, there is only one class named “fractured.”

The “Utilities” folder contains several notebooks used in the preparation of the dataset. As the manual annotations were done in COCO JSON format, the YOLO annotations were generated from the COCO masks using “coco2yolo.ipynb” and later PASCAL VOC annotations were generated from the YOLO annotations using “yolo2voc.ipnyb”. Under the subfolder “Fracture Split” there are 3 CSV files titled “test.csv”, “train.csv” and ‘valid.csv”. Each of these files contains a list of images used for testing, training and validation in the technical validation of the dataset respectively.

## Technical Validation

All the images in the dataset were manually examined to make sure no individually identifiable information is attached or embedded in the dataset images. After the annotation process of the dataset, all the masks generated were reviewed by the medical officer. To make sure the dataset is suitable for training machine learning algorithms we trained both fracture localization and segmentation using YOLOv8s and YOLOv8s-seg respectively.

The fractured images were randomly split into 80% (574) training, 12% (82) validation and 8% (61) test images for training and testing both the localization and segmentation models. The training was done on a Windows laptop equipped with an Nvidia RTX 3070 GPU with 8GB video memory and an AMD Ryzen 5900HX processor. Both the models were pre-trained with COCO40 and ran for 30 epochs. The input size for both cases was 600 pixels with standard hyperparameters. Table 2 lists the relative performance across different tasks.

**Table 2:** Result of baseline object (Fracture) detection and segmentation testing using pre-trained YOLO8s and YOLO8s-seg models on the FracAtlas dataset.

Task	Model	Type	Precision	Recall	mAP@0.5
Localization	YOLO8s	Box	0.807	0.473	0.562
Segmentation	YOLO8s-seg	Box	0.718	0.607	0.627
		Mask	0.83	0.499	0.589

### Object detection performance

For the localization task, the fractures were detected with a box precision of 80.7%, recall of 47.3% and an mAP of 56.2% at IoU of the 50th percentile on the validation set.

### Segmentation performance

For the segmentation task, the fractures were detected with a box precision of 71.8%, recall of 60.7% and an mAP of 62.7% at IoU of 0.5 on the validation set. As for the mask, the precision is 83%, recall 49.9% and mAP50 of 58.9%.

## Usage Notes

The dataset FracAtlas^19^ is made freely available for any purpose. The data provided within this work are free to copy, share or redistribute in any medium or format. The data might be adapted, remixed, transformed, and built upon. The dataset is licensed under a Creative Commons “Attribution 4.0 International” license (https://creativecommons.org/licenses/by/4.0/).

Additionally, any publication that utilizes this resource are requested to cite the original paper, and the authors are encouraged to share their code and models to help the research community reproduce the experiments and advance the field of medical imaging.

## Data Availability

The conversion of DICOM to JPEG image format was done using proprietary software of the X-ray machines from brands like Fujifilm and Philips hence they could not be made available. The mask annotations for segmentation were done using an open-source web tool named makedsense.ai. It was also used for generating VGG annotations from COCO format. As explained in the Methods section, the annotation conversion procedures from COCO to YOLO and YOLO to PASCAL VOC were performed using Python 3.10.1 on a Windows 11 operating system using ‘coco2yolo.ipynb’ and ‘yolo2voc.ipynb’. Both the Jupyter notebooks can be found inside the ‘Utility’ folder along with the dataset at Figshare (10.6084/m9.figshare.22363012). The code used for technical validation can be accessed from (https://github.com/XLR8-07/FracAtlas). There are 2 notebooks inside ‘notebooks’ under the root folder called ‘Train_8s.ipynb’ and ‘Prediction_8s.ipynb’. The ‘Train_8s.ipynb’ is used to train 2 models of ‘YOLO8s_seg’ and ‘YOLO8s’ variants targeted toward segmentation and localization tasks respectively. ‘Prediction_8s.ipynb’ is used to generate predictions out of the 2 aforementioned models and view the results.
